# Exploring psychedelic use in athletes and their attitudes toward psilocybin-assisted therapy in concussion recovery

**DOI:** 10.1177/20451253241264812

**Published:** 2024-08-07

**Authors:** Baeleigh VanderZwaag, Albert Garcia-Romeu, Mauricio A. Garcia-Barrera

**Affiliations:** Department of Psychology, University of Victoria, 3800 Finnerty Road, Victoria, BC V8P 5C2, CanadaInstitute on Aging and Lifelong Health, BC, Canada; Department of Psychiatry and Behavioral Sciences, Johns Hopkins University School of Medicine, Baltimore, MD, USA; Center for Psychedelic and Consciousness Research, Baltimore, MD, USA; Department of Psychology, University of Victoria, Victoria, BC, Canada; Institute on Aging and Lifelong Health, Victoria, BC, Canada

**Keywords:** athletes, mTBI, psilocybin, psychedelics, sports concussion

## Abstract

**Background:**

Psychedelics are receiving growing interest among clinical researchers for their effects on mood and cognition. Psilocybin is one of the most widely studied classic psychedelics which has shown good safety and clinical benefit for major depression and substance use disorders. Athletes frequently sustain concussions and often experience myriad symptoms, including cognitive and mood issues, which can persist for weeks or months in 10%–30% of athletes. Psilocybin may be a potential symptom management option for athletes with persisting concussion symptoms.

**Objectives:**

This study sought to summarize athlete psychedelic use, among other substances, and to examine the willingness of the sports community to engage in or support psilocybin-assisted therapy (PAT) for concussion recovery and management of persisting concussion symptoms.

**Methods:**

In total, 175 (*n* = 85 athletes; *n* = 90 staff) respondents completed an online survey distributed in Canada and the United States which queried sport involvement and demographics, substance use, concussion history, and knowledge and willingness about psilocybin. The reporting of this study conforms to the Checklist for Reporting Results of Internet E-Surveys (CHERRIES) statement.

**Design:**

Substance use rates were summarized across athletes and team staff members and a path analysis was used for each sample to identify predictors of willingness to use PAT (athletes) or support PAT (staff) for concussion recovery. Participants were also asked to identify perceived barriers to the implementation of PAT for sports-related concussions, and to indicate their overall willingness.

**Results:**

Psychedelics were the third most used substance in the past year among athletes (35.8%) while regular psychedelic use was quite low in athletes (7.5%). A path analysis conducted in RStudio found that attitudes toward psilocybin and knowledge of psilocybin were significant predictors for both athletes and staff members of their willingness to use or support PAT for concussion recovery. Athletes reported likely engaging in PAT (61.2%) and staff (71.1%) reported that they would support their athletes using PAT.

**Conclusion:**

The results of this study suggest that the sports community may be receptive to PAT and athletes would be willing to engage in it for concussion recovery and/or the management of persisting post-concussion symptoms (PPCS). Future research should examine the effects of psilocybin for PPCS to inform whether there is any impact while addressing concerns regarding long-term effects of psilocybin use.

## Plain language summary

Psilocybin-assisted therapy for concussion recovery

**Study purpose:** Psychedelics such as psilocybin have been studied largely in patients with terminal cancers, but research has begun expanding to the mental health field and for cognitive benefit. Athletes who sustain concussions and experience persisting concussion symptoms are at an increased risk of developing symptoms of anxiety and depression and dealing with cognitive symptoms such as impaired memory, attention, and concentration. Current research with psilocybin suggests that examining the use of psilocybin in athletes post-concussion for the management of mood and cognitive symptoms is a worthwhile endeavour. However, it is unclear whether the sports community is receptive to this line of research.

**What did the researchers do?** We employed a survey study to athletes and sports staff across Canada and the United States to examine current psychedelic use, motivations for use, and willingness to use or support psychedelic-assisted therapy (PAT) in athletes post-concussion.

**What did the researchers find?** 175 respondents completed an online survey distributed in Canada and the United States (n = 85 athletes; n = 90 staff). Psychedelics were the third most used substance in the past year among athletes (35.8%) while regular psychedelic use was quite low in athletes (7.5%). A path analysis conducted found that attitudes towards psilocybin and knowledge of psilocybin were significant predictors for both athletes and staff members of their willingness to use or support PAT for concussion recovery. Athletes reported likely engaging in PAT (61.2%) and staff (71.1%) reported that they would support their athletes using PAT.

**What do these findings mean?** Our study suggests that athletes may be willing to use PAT if they had sustained a concussion and were dealing with persisting symptoms while their coaches and other staff members would also be willing to support them. Thus, further research through randomized clinical trials will be necessary to understand the effects of psilocybin and other psychedelics on persisting concussion symptoms in athletes.

## Background

Sports-related concussions (SRC) are a form of mild traumatic brain injury (mTBI) that occurs while engaging in sports.^1^ In the United States, roughly 3.8 million sports-related brain injuries occur annually^2^ while in Canada, available data from 2020 to 2021 identified almost 2000 emergency department visits for sports-related brain injuries.^3^ The conceptual definition of SRC according to the Concussion in Sport Group (CISG) was updated at the Amsterdam 2022 International Consensus Statement on Concussion. This improved definition involves the following major points: (1) force is transmitted to the brain via a blow to the head, neck, or body through sport or sport-related activities; (2) the resulting injury to the brain occurs through a neurotransmitter and metabolic cascade; (3) symptoms can onset rapidly or overtime and typically resolve within days but may persist; (4) injury is typically functional rather than structural and will not be seen on standard structural neuroimaging studies; and (5) clinical presentation is broad and diverse and may or may not include loss of consciousness.^4^ Therefore, SRCs can be understood as a type of mTBI which are a serious injury that can significantly affeet quality of life. While most athletes fully recover within 7–10 days, approximately 10%–30% of athletes will experience persisting post-concussion symptoms (PPCS) involving long-term persistence (for weeks or even months) of clinical symptoms past this typical recovery time frame.^5–8^ Common psychological symptoms include mood dysregulation (i.e. anxiety, depression, irritability) and cognitive impairments in executive function, memory, attention, and concentration.^6,9,10^ Physiological symptoms include headache, fatigue, and light and noise sensitivity.^6^ Both psychological and physiological symptoms can be greatly debilitating in athletes across multiple areas of life (i.e. interpersonal, academic, work, leisure, sport) and can prolong the time before returning to work, school, and/or sport.^5^ Currently, the recommended management of symptoms from the Canadian Guideline on Concussion in Sport includes cognitive and physical rest^11^ which may be supplemented by nonsteroidal anti-inflammatory drugs in the acute phase of concussion recovery.^12^ This rest period is followed by a step-wise progression to reintroduce regular activities beginning with light cognitive load until full return to activity is achieved.^11^ However, despite this approach being the primary treatment recommendation, this approach does not work for every athlete, and because up to 30% may endure PPCS, an individualized treatment approach must be implemented. Improved concussion rehabilitation approaches and managing PPCS are among the most requested areas of research from patients, clinicians, and researchers.^4,13^ Therefore, the current study explores the willingness of the sports community to engage in (as an athlete) or support (as a staff member) psilocybin-assisted therapy (PAT) for concussion recovery and PPCS in athletes. This study aims to contribute to the ongoing discourse surrounding concussion rehabilitation and offer insights into novel avenues such as psychedelic therapy for managing PPCS.

## The role of psilocybin

Psilocybin is a classic psychedelic that is metabolized into psilocin on ingestion.^14^ Research suggests that activity at the serotonin 2A receptor (5-HT_2A_R) is a key mechanism of the psychedelic effects of psilocybin.^15^ Psilocybin has grown in popularity in recent years among researchers in an era termed the “psychedelic renaissance.”^16–18^ Researchers worldwide have examined psilocybin’s effects on mental health in a myriad of conditions such as treatment-resistant depression, end of life anxiety, and substance use disorders.^19–22^ A recent scoping review summarized the effects of psilocybin on cognitive functioning, finding temporally variable effects, suggesting that although psilocybin may lead to acute cognitive impairment, it does not impair cognitive functioning long-term.^23^ Notably, psilocybin was rarely found to result in severe adverse side effects, and potential for abuse or toxicity are low, indicating good safety when administered to carefully screened and prepared individuals in controlled settings.^14,23–25^ This safety profile is especially important when considering use in clinical populations with a brain injury.

Although psilocybin has not been formally investigated in people with SRC, we hypothesize that psilocybin may benefit those with sports concussion and persisting symptoms through three primary mechanisms: (1) acting as an anti-inflammatory agent via 5-HT_2A_R to limit prolonged neuroinflammation^26,27^; (2) inducing neurogenic effects in brain regions implicated in cognitive functioning^28,29^; and/or (3) by managing symptoms of anxiety and depression which are frequent after SRC and particularly in those presenting with PPCS.^20,21,30–32^

## The current study

While current research highlights a theoretical proof of concept in examining PAT for SRC and PPCS,^23,26,27,29,30,31,33^ it is unclear whether the sports community may be receptive to the use of psilocybin in concussion recovery. Data from a U.S. national survey found that adults aged 26–34 were more likely to engage in psilocybin use compared to adults 18–25 years old, and respondents who completed high school and/or college were more likely to report having used psilocybin.^34^ In a sample of psychotherapists, attitudes and knowledge of psilocybin were positively correlated with willingness to recommend PAT to patients.^35^ Similarly, Melnikov et al.^36^ found that attitudes toward medical cannabis significantly influenced the likelihood that healthcare professionals would recommend medical cannabis as a treatment to their patients. Thus, variables such as age, education, attitudes, and knowledge emerge as important variables to examine in one’s willingness to engage in or support PAT for concussion recovery. Therefore, this study was designed to gather relevant information to provide an understanding of interest and concerns from athletes, coaches, and other sports staff regarding the therapeutic use of psilocybin.

This study used a descriptive, open, online survey approach to address the following three objectives. The first objective is to provide an estimate of current rates of general psychedelic use among athletes in Canada and the United States across levels of competition and the motivations for use. The second objective is to summarize the sports community’s current attitudes and beliefs specifically about psilocybin and its therapeutic use for concussion recovery and managing persistent concussion symptoms. The third objective is to report the willingness of athletes to engage in psilocybin for the treatment of concussion and persistent symptoms and the level of support from sports team personnel for athletes interested in this treatment option.

Given the lack of research on this topic, we will examine current psychedelic use rates among athletes and motivations for use in athletes using an exploratory approach. Based on previous literature, it is hypothesized that greater age, education, knowledge, and attitudes will predict willingness to engage in or support PAT, and we examine concussion history as an exploratory variable in the athlete sample.

## Methodology

### The reporting of this study conforms to the CHERRIES statement^37^

#### Survey features

All respondents were required to complete a Captcha verification (Completely Automated Public Turing Test to tell Computers and Humans Apart) to ensure unique visitors and human participants, prior to beginning the informed consent process.^37^

Using skip logic, questions were only presented to respondents when they met criteria for that question (e.g. by selecting “athlete,” further questions on concussion history were revealed), to prioritize response efficiency. Respondents were requested to complete all questions but were not required to do so to submit their survey response. Due to the anonymized nature of the survey, we did use cookies, track IP addresses, or require respondents to log in to access the survey.

#### Participants

For this study, the population of interest included athletes and individuals involved as staff members to a sport team at any capacity (i.e. coach, trainer, or physical therapist) and at any level of competition (i.e. recreational, club, collegiate, etc.). Inclusion criteria were: (1) current involvement in a sports team as an athlete or a team personnel; (2) 18 years old or older; and (3) currently living within Canada and the United States. This study involved the completion of a three-part online survey. Exclusion criteria were thus: (1) not involved in sports; (2) under the age of 18 years old; and (3) currently living outside of Canada and the United States.

#### Survey section 1: demographics and clinical information

Key demographic information was collected including age, gender, race/ethnicity, country, sportsmanship level, sport(s) currently engaged in, and role (athlete or team personnel). The Ten-Item Personality Inventory^38^ was used to examine the relationship between Big Five personality traits and the primary study variables. Finally, concussion history was collected from athletes which included questions about history of concussion, diagnosis, symptoms experienced, and what symptoms, if any, persisted after returning to sport.

#### Survey section 2: substance use information

Information was collected from athletes pertaining to history of and current psychedelic use in addition to other drugs/alcohol. Questions included history and current use, psychedelic(s) of choice, method of intake, and motivations for use (mood enhancement, cognitive enhancement, pain management, relaxation, coping, etc.).

#### Survey section 3: attitudes and beliefs toward psilocybin

Information was collected from athletes and sports team personnel regarding attitudes and beliefs toward psilocybin and willingness to incorporate it in PPCS recovery. Both the team personnel group and athlete group were asked about World Anti-Doping Agency regulation of psilocybin, attitudes toward psilocybin, knowledge of psilocybin, beliefs about addictive properties, perceived benefit of research on the topic, interest in learning more about psilocybin for medical use, and barriers to the implementation of PAT for concussion recovery in athletes. Athletes were queried on their willingness to *use PAT* for concussion recovery while staff were queried on their willingness to *support athletes using PAT*. Questions were ranked using a 7-point Likert scale. The full survey can be found in Supplemental Materials.

#### Sampling methods

Our sample was recruited using a variety of methods to acquire a diverse sample base. These methods included distribution of study flyers online and via social media pages specific to sports and athletes, such as Reddit forums frequented by sport participants, distribution of study information to university and college athletic departments, and word of mouth snowball sampling among athletes and training staff. Respondents were entered to win one of four gift cards following completion of the survey. E-mail addresses requested for entering the raffle were acquired through a redirected survey to separate addresses from survey responses and were deleted after the raffle draw was completed.

### Statistical analyses

Descriptive statistics (i.e. mean, standard deviation, skew, kurtosis) were calculated for the entire sample as well as each category (athletes/team personnel). Data were examined for outliers using the Mahalanobis distance. To limit accidental omission of responses, we employed a request response validation if a question was not answered, with the option to skip the question if participants chose not to answer.

Two path analyses were conducted in RStudio version 2021.09.0 to test two proposed models (one for athletes and one for staff) developed based on prior literature and exploratory variables such as concussion history (for the athlete model only). The proposed models (see Supplemental Materials) were developed to test the effect of demographic variables (age and education), personality trait openness (measured through the Ten-Item-Personality Inventory [TIPI]), knowledge of psilocybin, attitudes toward psilocybin, past psychedelic use experience, and concussion history (for the athlete model) on willingness to engage in or support PAT for persisting concussion symptoms. All other data analysis was conducted in SPSS Version 27 (IBM Corp. Released 2020. IBM SPSS Statistics for Windows Version 27.0 Armonk, NY: IBM Corp).

## Results

Missing data patterns were assessed using Little’s Missing Completely at Random (MCAR) analysis. Results for athletes and staff indicated that missing data (<1%) can be assumed to be MCAR with no systematic pattern of missingness (χ^2^ = 6.058, df = 6, *p* = 0.417; χ^2^ = 8.785, df = 11, *p* = 0.642).

### Overall respondent characteristics

At the end of the data collection period (June 1, 2023), our sample consisted of 175 participants, out of 235 initiated surveys (74% completion rate; Table 1), a majority of which were male (67.4%), Caucasian (59.4%), and from the United States (57.7%) between the ages of 18 and 76 years old, with a mean (*M*) age of 30.52 years (SD = 9.02). Demographic information are described separately for the athlete and staff samples below and demographics for the full sample can be found in Table 1.

**Table 1.** Characteristics of participants.

Characteristic	Athlete sample, *n* (%) and/or *M* (SD)	Staff sample, *n* (%) and/or *M* (SD)	Full sample, *n* (%) and/or *M* (SD)
Age	85 (48.6%)/28.73 (9.65)	90 (51.4%)/32.21 (8.07)	175/30.52 (9.02)
Gender			
Male	59 (69.4%)	59 (65.6%)	118 (67.4%)
Female	24 (28.2%)	30 (33.3%)	54 (30.9%)
Other	2 (2.4%)	1 (1.1%)	3 (1.7%)
Marital status			
Single	44 (51.8%)	32 (35.6%)	76 (43.7%)
Married	41 (48.2%)	57 (63.1%)	98 (56.3%)
Education			
High school	3 (3.5%)	1 (1.1%)	4 (2.3%)
Some college or university	24 (28.2%)	16 (17.8%)	40 (22.9%)
Bachelor’s degree	33 (38.8%)	45 (50.0%)	78 (44.6%)
Master’s degree or equivalent	14 (16.5)	20 (22.2%)	34 (19.4%)
Doctoral degree or equivalent	9 (10.6%)	8 (8.9%)	17 (9.7%)
Certificate program	1 (1.2%)	0 (0.00%)	1 (0.6%)
Other	1 (1.2%)	0 (0.00%)	1 (0.6%)
Ethnicity^a^			
White	58 (65.2%)	46 (43.4%)	104 (59.4%)
Latinx/Hispanic	12 (13.5%)	16 (15.1%)	29 (16.6%)
Indigenous	14 (15.7%)	14 (13.2%)	28 (16.0%)
Black	1 (1.1%)	8 (7.5%)	9 (5.1%)
East Asian	2 (2.2%)	5 (4.7%)	7 (4.0%)
Filipino	1 (2.2%)	5 (4.7%)	7 (4.0%)
Southeast Asian	0 (0.00%)	5 (4.7%)	5 (2.9%)
South Asian	0 (0.00%)	3 (2.8%)	3 (1.7%)
West Asian or North African	0 (0.00%)	3 (2.8%)	3 (1.7%)
Middle Eastern	0 (0.00%)	1 (0.9%)	1 (.6%)
Country			
Canada	41 (48.2%)	33 (36.7%)	74 (42.3%)
United States	44 (51.8%)	57 (63.3%)	101 (57.7%)
Lifetime drug use			
Yes	53 (62.4%)	36 (40.0%)	89 (50.8%)
No	30 (35.3%)	52 (57.8%)	82 (46.8%)
Prefer not to say	2 (2.4%)	2 (2.2%)	4 (2.4%)
Regular drug use^a,b^			
Alcohol	27 (50.9%)	18 (52.9%)	45 (51.7%)
Cannabis	10 (18.9%)	10 (29.4%)	20 (23.0%)
Tobacco	8 (15.1%)	11 (32.4%)	19 (21.8%)
Smokeless tobacco	1 (1.9%)	2 (5.9%)	3 (3.4%)
Opiates	1 (1.9%)	1 (2.9%)	2 (2.3%)
Psychedelics	4 (7.5%)	2 (5.9%)	6 (6.9%)
Stimulants	0 (0.00%)	2 (5.9%)	2 (2.3%)
Anabolic steroids	2 (3.8%)	1 (2.9%)	3 (3.4%)
None	10 (18.9%)	8 (23.5%)	18 (20.7%)
Other (ketamine)	1 (1.9%)	0 (0.00%)	1 (1.1%)
Sport level^a^			
Recreational	41 (33.9%)	22 (16.1%)	41 (33.9%)
Club	26 (21.5%)	42 (30.7%)	26 (21.5%)
Collegiate/varsity	37 (30.6%)	34 (24.8%)	37 (30.6%)
National	9 (7.4%)	17 (12.4%)	9 (7.4%)
Professional	8 (6.6%)	22 (16.1%)	8 (6.6%)
Concussion history			
Yes	48 (56.5%)		
No	34 (40.0%)		
Don’t know	3 (3.5%)		
Number of concussions^c^			
1	15 (17.6%)		
2	9 (10.6%)		
⩾3	22 (25.9%)		
Don’t know	2 (2.4%)		
None	37 (43.5%)		
Number of concussion symptoms^c,d^			
1–4	31 (64.6%)		
5–9	13 (27.1%)		
⩾10	3 (6.3%)		
None	1 (2.1%)		
Number of persisting concussion symptoms^c,d^			
1	22 (45.8%)		
2	9 (18.8%)		
⩾3	9 (18.8%)		
None	8 (16.7%)		

a Participants could select more than one option.

b Indicates questions responded to only by participants who reported using drugs or alcohol in the past year.

c Indicates questions responded to only by athletes (*n* = 85).

d Answered only by athletes with a history of concussion (*n* = 48).

### Athlete demographics

Our athletes sample consisted of 85 athletes (*M*_Age_ = 28.73, SD = 9.65) with most athletes from the United States (51.8%). Most athletes (65.2%) identified as Caucasian and most frequently played American football (17.6%), softball (17.6%), volleyball (16.5%), and soccer (11.8%). Most athletes played recreational (*n* = 41) and collegiate (*n* = 37) sports.

### Staff demographics

Our staff sample consisted of 90 staff members (*M* = 32.21, SD = 8.07) who primarily identified as male (65.6%) from the United States (63.3%). Staff members were most frequently coaches (53.3%) at the collegiate level (38.2%) and the most common sport they coached was softball (22.2%).

### Drug use findings

#### Overall

Just over half of our sample (50.8%) indicated using drugs or alcohol at some point in their life with the most frequent substance used among these respondents being alcohol (95.5%) followed by cannabis (55.1%) and psychedelics (41.6%). Over the past year, 87 participants (49.7%) indicated using drugs or alcohol, of which most used alcohol (93.1%), cannabis (43.7%), and psychedelics (34.5%). Finally, 71 participants (40.6%) indicated regularly using drugs or alcohol (⩾2 times per week), most commonly alcohol (51.7%), cannabis (23.0%), and tobacco (21.8%).

#### Athletes

Most athletes indicated using drugs at least once in their life (62.4%) while two athletes selected to not disclose their drug use history. Of those who reported using drugs at some point in their life (*n* = 53), all reported using drugs or alcohol within the past year and 56.5% of athletes indicated using drugs or alcohol regularly. The most commonly used substances in the past year were alcohol (90.6%), cannabis (43.4%), and psychedelics (35.8%) while the most commonly used substances regularly (⩾2 times per week) were alcohol (50.9%), cannabis (18.9%), and tobacco (15.1%). With regard to the first objective of this study, to provide an estimate of psychedelic use among athletes, we found that 22.4% of athletes reported using psychedelics in their lifetime. Among those, 35.8% reported using psychedelics in the past year, and 7.5% reported using psychedelics regularly. The most common psychedelic used among athletes was psilocybin (64.3%). The primary motivations for using psychedelic among athletes was personal improvement or general well-being (16.4%) and mood enhancement (12.3%). As previously mentioned, the majority of our athlete sample competes at recreational and collegiate levels. Therefore, these rates of psychedelic use may not apply to athlete populations with more rigid substance use restrictions.

#### Psychedelic use among athletes and staff

All respondents who reported using psychedelics within the past year (34.5%) were asked about their patterns and reasons of use. Our participants primarily indicated using psychedelics a few times a year with the most common psychedelic used being psilocybin (59.6%). The reasons for use were most often for personal improvement (14.5%) and mood enhancement (13.6%). Participants reported using psilocybin for a number of health-related conditions including anxiety (*n* = 16), depression (*n* = 16), and trauma-related reasons (*n* = 9). Participants generally reported improvements in these areas.

### Concussion history frequency

Our survey found that 56% (*n* = 48) of our athletes had experienced at least one concussion. Of those athletes, 83.3% reported receiving a medical diagnosis of a concussion with 90% of these concussions being diagnosed by a physician. The most frequently endorsed concussion symptoms were headache (13.4%), nausea (10.9%), and fatigue (10.5%). Athletes reported myriad symptoms which could be captured under the umbrella term of “cognitive symptoms.” These cognitive symptoms include feeling in a fog, feeling slowed down, memory complaints, and difficulties with concentration and attention. A total of 29.3% of reported symptoms among athletes fell within this umbrella of “cognitive” symptoms. Mood symptoms accounted for 6.1% of those experienced by athletes.

In our sample of athletes, persisting concussion symptoms occurred in 83% (*n* = 40) of those who reported a history of concussion. Persisting symptoms lack a strong definition given poor consensus among the field. However, we asked athletes which symptoms persisted after they were cleared to reengage in sports performance. The most frequently reported persisting symptoms included headache (21.7%), nausea (10.8%), fatigue (10.8%), and sleep difficulties (10.8%). Again, cognitive symptoms were highly reported with 21.7% of persisting symptoms being cognitive while mood symptoms were less likely to persist (4.8%).

### Attitudes toward psilocybin

#### Athlete attitudes

Just over half of the athlete sample was familiar to some degree with psilocybin (56.5%) and 38.8% had favorable attitudes toward psilocybin while 40% had some level of knowledge of psilocybin. Most important, 67.1% of athletes believe that psilocybin was addictive or had the ability to be abused. However, over half of athletes were interested in learning more about PAT for mental health, concussion recovery, and other medical concerns (55.3%) and felt that there was benefit in examining the medical use of psilocybin (57.6%).

#### Staff attitudes

Just over half of team staff were familiar with psilocybin (54.4%), had favorable attitudes toward psilocybin (52.2%), and were knowledgeable of psilocybin (54.4%). Over half of the team staff felt that psilocybin was addictive or likely to be abused (57.8%). Two-thirds of staff felt that there was benefit in examining the medical uses of psilocybin (66.7%) and the majority of staff were interested in learning more about the medical uses of psilocybin (76.7%).

### PAT willingness

#### Athlete willingness

**Figure 1.**
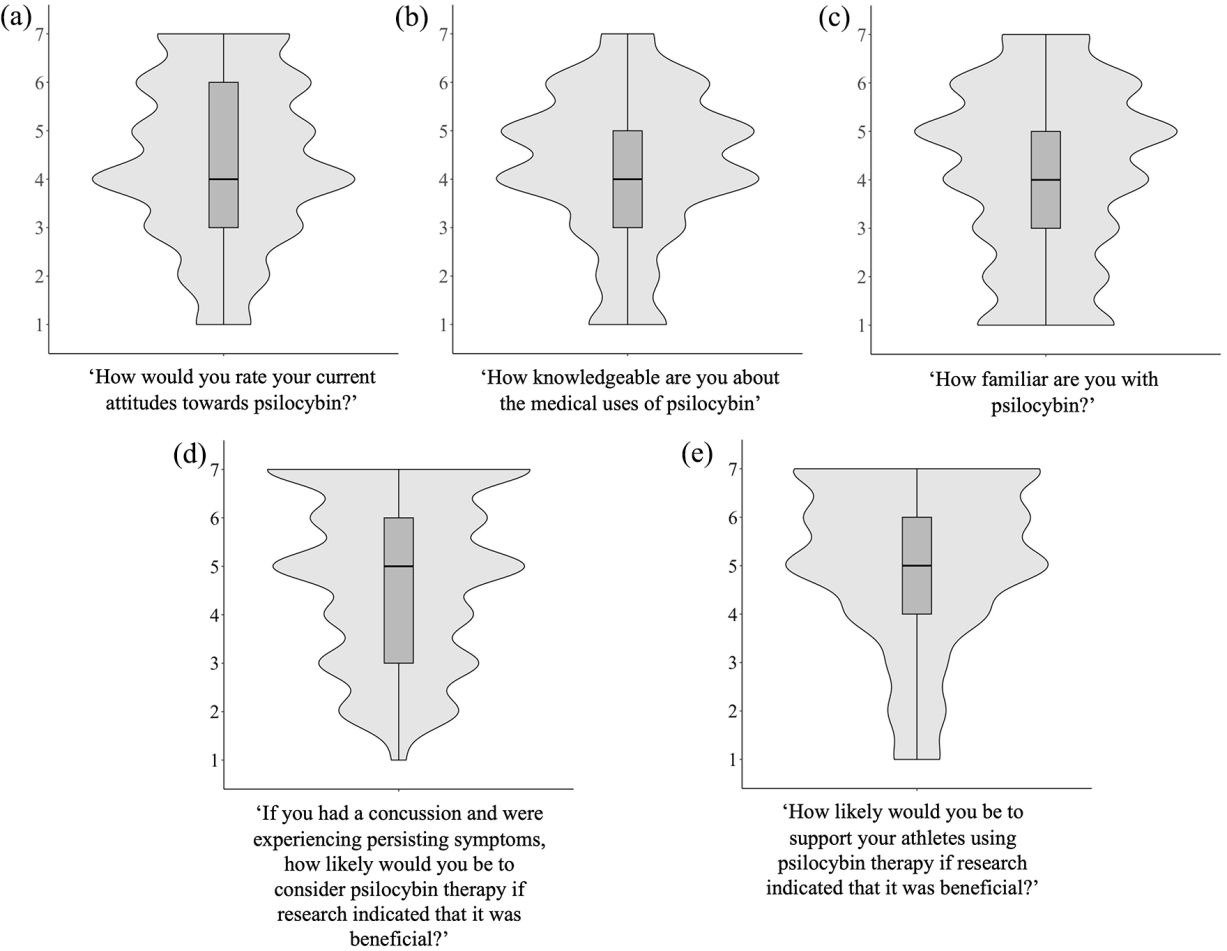
Violin plots displaying the density-based distribution of the Likert scale options for (a) attitudes toward psilocybin (all participants), (b) knowledge of psilocybin (all participants), (c) familiarity with psilocybin (all participants), (d) willingness to use PAT if you had a concussion (athletes only), and (e) willingness to support athletes using PAT for concussion recovery (staff only).

Athletes were asked whether they would be willing to engage in PAT for PPCS if they were experiencing it themselves and research indicated that PAT was beneficial for this purpose. About a quarter (23.5%) of athletes indicated that they would be “very likely” to engage in PAT. In addition, 22.4% and 15.3% indicated that they would be “likely” and “somewhat likely,” respectively, to engage in PAT for this purpose (Figure 1(d)). Finally, 25.9% of athletes indicated that they would be “very unlikely,” “unlikely,” or “somewhat unlikely” to engage in PAT for concussion recovery.

#### Staff willingness

Sports team staff were asked whether they would be willing to support their athlete(s) dealing with PPCS in using PAT if research indicated that it was beneficial, with 25.6% of staff indicating that they would “likely” support this, while 21.1% indicated they would be “somewhat likely,” and 24.4% “very likely.” Another 15.6% of sports team staff indicated that they would be “very unlikely,” “unlikely,” or “somewhat unlikely” to support PAT for concussion recovery (Figure 1(e)).

### Perceived barriers to PAT for concussion recovery

We queried athletes and staff regarding significant barriers to implementing PAT for concussion recovery in athletes. A recurrent theme across athletes and staff were concerns regarding the long-term effects of psilocybin therapy with 24.0% of athletes and 24.7% of staff indicating this as a concern. Athletes highlighted the stigma from their coaches or other team staff (18.3%) as another prominent concern whereas staff believed access to psilocybin treatment (19.2%) to be a significant barrier.

### Path analysis

#### Correlations

Pearson correlations were conducted with the path variables of interest to examine any significant relationships, direction of relationships, and any especially strong relationships (i.e. multicollinearity). This was especially necessary for exploratory variables. Correlation coefficients revealed that willingness to use PAT among athletes was significantly and positively correlated with psilocybin attitudes (*r* = 0.57, *p* < 0.001) and psilocybin knowledge (*r* = 0.55, *p* < 0.001). Willingness to support PAT as a staff member was also significantly and positively correlated with psilocybin attitudes (*r* = 0.48, *p* < 0.001) and psilocybin knowledge (*r* = 0.49, *p* < 0.001). All other correlations were not statistically significant, see Supplemental Materials for full correlation results.

#### Athlete model

**Figure 2.**
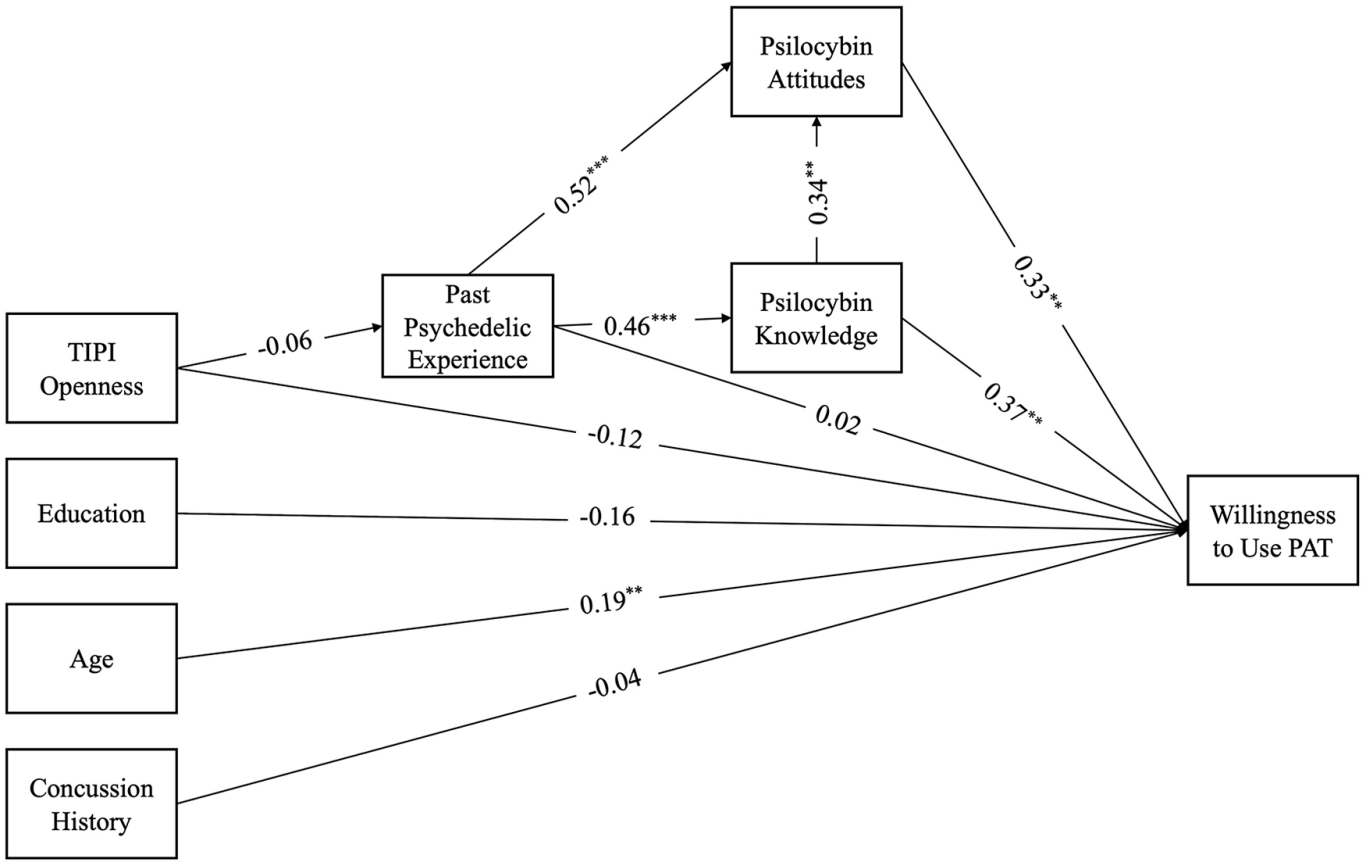
Path model with standardized coefficients for athletes. *p < 0.05. **p < 0.01. ***p < 0.001.

The model fit indices indicated a good fit to the data, χ^2^ = 139.19, df = 22, *p* < 0.001, Comparative Fit Index (CFI) = 0.98, Tucker-Lewis Index (TLI) = 0.96, Root Mean Square Error Approximation (RMSEA) = 0.05. The results revealed significant direct paths from age to willingness (β = 0.19, SE = 0.01, *p* < 0.01), knowledge to willingness (β = 0.37, SE = 0.14, *p* < 0.01), from attitudes to willingness (β = 0.33, SE = 0.11, *p* < 0.01), attitudes to knowledge (β = 0.34, SE = 0.12, *p* < 0.01), attitudes to psychedelic experience (β = 0.52, SE = 0.38, *p* < 0.001), and knowledge to psychedelic experience (β = 0.46, SE = 0.33, *p* < 0.001). Past psychedelic use, concussion history, and education were not significant predictors of willingness. These findings suggest that higher levels of knowledge of psilocybin are associated with more positive attitudes toward psilocybin as well as greater willingness to use PAT. There was a significant indirect effect between past psychedelic experience and willingness (β = 0.17, *p* < 0.01) suggesting that knowledge mediates the relationship between these two variables. Similarly, there was a significant indirect effect between past psychedelic experience and attitudes (β = 0.16, *p* < 0.01) with knowledge as a mediator (Figure 2).

#### Staff model

**Figure 3.**
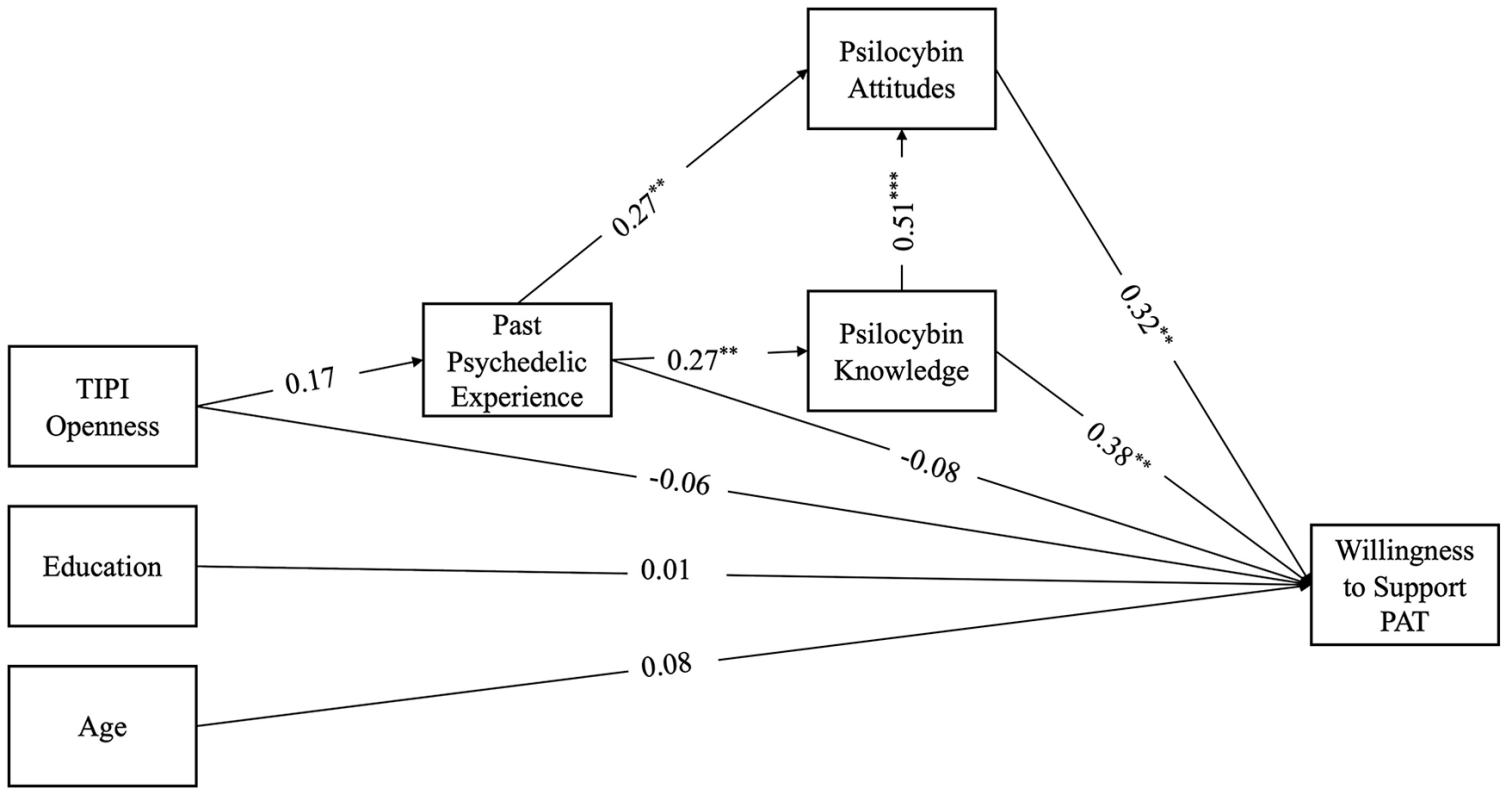
Path model with standardized coefficients for staff. *p < 0.05. **p < 0.01. ***p < 0.001.

The model fit indices indicated a good fit to the data, χ^2^ = 99.51, df = 18, *p* < 0.001, CFI = 0.99, TLI = 0.97, RMSEA = 0.04. The results revealed significant direct paths from knowledge to willingness (β = 0.32, SE = 0.12, *p* < 0.01), attitudes to willingness (β = 0.32, SE = 0.11, *p* < 0.01), from attitudes to knowledge (β = 0.51, SE = 0.09, *p* < 0.001), attitudes to past psychedelic experience (β = 0.27, SE = 0.38, *p* < 0.01), and knowledge to psychedelic experience (β = 0.27, SE = 0.38, *p* < 0.01). There was a significant indirect effect between past psychedelic experience and attitudes (β = 0.14, *p* < 0.05) with knowledge as a mediator as well as a significant indirect effect between past psychedelic experience and willingness (β = 0.16, *p* < 0.01) mediated by knowledge (Figure 3).

## Discussion

Psychedelic use by athletes has been scarcely documented, and to our knowledge, this is the most comprehensive and recent examination of psychedelic use in Canadian and American athletes. This is also the first survey to examine athlete willingness to engage in PAT for concussion recovery and persisting concussion symptoms and staff willingness to support this treatment in athletes. Despite researchers discussing the clinical utility of psilocybin for athlete mental health, particularly in those with a history of concussion,^39^ research on this population has not been yet published. The use of psychedelics for mental health concerns in former athletes is being discussed in mainstream news with popular athletes such as former National Hockey League players Daniel Carcillo and Riley Cote coming forward to discuss their experiences with psilocybin.^40^ These anecdotal experiences suggest the examination of psilocybin use in concussion recovery and persisting symptom management is a timely and worthwhile endeavor, and this study may begin to pave way for the examination of psilocybin for acquired brain injury. Most important, the implementation of psilocybin for the management of PPCS would strongly depend on the willingness of the population to engage in this approach, thus this study represents an important early step prior to the commencement of clinical trials.

We found some evidence of current psychedelic use among athletes across levels of competition and that this sample of athletes favors psilocybin. Our sample primarily consisted of recreational and collegiate athletes with limited professional athlete respondents. Compared to a previous study in American collegiate athletes, the only other survey to discuss athlete psychedelic use, we identified higher rates of psychedelic use in our sample.^41^ This may in part be due to the rising discussion of psychedelics among researchers and mainstream media. Moreover, growing discourse among former elite athletes may also fuel the increase of psychedelic use among our sample of athletes. In contrast to prior literature, the primary reasons athletes in our sample used psychedelics were for personal improvement and mood enhancement, compared to recreational or social reasons.^41^

While our sample endorsed primarily neutral attitudes toward psilocybin, respondents were somewhat familiar with psilocybin and knowledgeable about the medical uses of psilocybin according to self-identified knowledge and familiarity. However, given that these were self-reported, there is potential for biases affecting their ratings, and their responses may not be supported by accurate knowledge. Specifically, staff were more likely to be concerned about possible addictive properties of psilocybin or the potential misuse than athletes were, despite research largely refuting the addictive potential of classic psychedelics.^42^ Most important, both athletes and coaches were concerned about the long-term effects of using psilocybin. Last, most participants were interested in learning more about psilocybin’s potential use in mental health management and believed that there is benefit in examining psilocybin’s use in PPCS management.

Findings from this survey suggest that our athlete population may be receptive to PAT through concussion recovery and the management of PPCS even in the presence of generally neutral attitudes toward psilocybin. This openness suggests viability of clinical research with an athlete sample to examine effects of psilocybin in PPCS, specifically examining the effects on the cognitive and mood symptoms. Provided that nearly a quarter of our sample endorsed cognitive symptoms following their concussion and 6.1% endorsed mood dysfunction, it is worth examining whether psilocybin may offer relief for athletes who experience such symptoms.

While research with other clinical populations (i.e. treatment-resistant depression, cancer-related distress, substance use disorders) suggest therapeutic benefits of psilocybin, it is unclear whether these outcomes would translate in patients with brain injury. Similarly, some research indicates cognitive improvement with psilocybin use or negligible cognitive outcomes, but many studies which examine these cognitive outcomes have often used healthy participants rather than clinical populations,^23^ limiting generalizability to TBI patients. Nonetheless, long-term outcomes of psilocybin represent a significant gap in the literature and were primary concerns of both athletes and staff regarding PAT in athletes with concussions.

As shown in both path analysis models, knowledge about psilocybin plays a significant role in willingness to use or support PAT. Specifically, higher levels of knowledge of psilocybin were associated with more positive attitudes toward psilocybin as well as greater willingness to use and support PAT. By identifying the impact of knowledge on willingness for both athletes and staff, recommendations can be made to improve knowledge dissemination to the sports community regarding psilocybin’s safety and risks, current understanding regarding effects on athletic performance, and proposed effects that psilocybin might have in helping manage PPCS. This knowledge dissemination to and broad discussion with those that this treatment approach intends to benefit will be paramount. These findings also highlight the importance of taking a *patient-oriented approach* in future research which will involve athletes and staff in the research process. Their involvement will inform research design, development of treatment protocols, considerations for clinical implication, and concerns regarding sports performance and other long-term effects. Thus, we can ensure that the sports community’s voice is heard, and that the results be more efficiently applied to those in need.

### Limitations

It is important to acknowledge certain limitations of the present study. First, the reliance on self-reported data invites the potential for response bias which could lead participants to respond inaccurately or in a socially desirable manner. When surveying participants on drug use history and experiences, athletes may be hesitant to report their true experiences due to fears of potential consequences for drug use. It is also worth highlighting that bias may occur when participants rate their knowledge or proficiency in a subject. Thus, our measure of self-reported knowledge may not reflect accuracy in knowledge. Second, it is worth noting that athletes and sports team staff who responded to this survey may have done so out of personal interest or prior experience with psychedelics, potentially introducing a sample bias and inflated psychedelic use rates among athletes. We also recognize the difficulty in recruiting professional athletes, and that our sample lacked sufficient input from these athletes. Receptivity of PAT from such a population may differ based on rates of concussions among professional athletes, differences in training and intensity of play, and overall accessibility to psychedelics for a therapeutic purpose. Third, our survey found that almost half of our participants reported being drug naïve (i.e. never having used drugs or alcohol before). While we provided a brief description of psilocybin that avoided any bias (“The following questions focus on psilocybin which is a naturally occurring psychoactive substance found in Psilocybe mushrooms, also known recreationally as “magic mushrooms”), additional information may have been necessary to provide drug naïve participants with a more comprehensive base for what psilocybin is for the next questions. As such, neutral responses may have been a result of a limited understanding of what psilocybin is and/or the current state of psilocybin research.

## Conclusion

The findings of this study suggest a high level of receptiveness in the sports community toward using and supporting PAT for concussion recovery given evidence that it is beneficial. These findings highlight the feasibility of collaborating with the sports community to examine this innovative therapeutic approach. Most important, knowledge about psilocybin emerges as a crucial factor influencing willingness, underscoring the importance of future initiatives that focus on fostering discourse between the scientific and sports communities. In bridging the gap between the communities and working in a collaborative approach, researchers can work to address key barriers identified by both athletes and staff members. Overall, this study indicates that conducting clinical research with athletes suffering from SRC and PPCS is a valuable research endeavor.

## Supplemental Material

sj-docx-1-tpp-10.1177_20451253241264812 – Supplemental material for Exploring psychedelic use in athletes and their attitudes toward psilocybin-assisted therapy in concussion recovery Supplemental material, sj-docx-1-tpp-10.1177_20451253241264812 for Exploring psychedelic use in athletes and their attitudes toward psilocybin-assisted therapy in concussion recovery by Baeleigh VanderZwaag, Albert Garcia-Romeu and Mauricio A. Garcia-Barrera in Therapeutic Advances in Psychopharmacology

## Declarations

**Ethics approval and consent to participate:** The present study received ethics approval (H22-03498) from the Research Ethics Boards at the University of Victoria and the University of British Columbia through the harmonized ethics review process. The study was conducted in accordance with the ethical guidelines set forth by the Tri-Council Policy Statement: Ethical Conduct for Research Involving Humans. This study was preregistered through Open Science Framework on March 13, 2023, before data collection commenced.^43^ All participants provided electronically signed consent for participation after reading the consent form.

**Consent for publication:** Not applicable.

**Competing interests:** AG-R is a paid scientific advisor to NeonMind Biosciences and InnerWell. AG-R has received research funding from MicroDoz Therapy Inc., Mydecine Innovations Group Inc., Unlimited Sciences, the Council on Spiritual Practices, the Heffter Research Institute, and the National Institutes of Health. Effort from AG-R on this work was supported through the Johns Hopkins Center for Psychedelic and Consciousness Research with funding provided by Tim Ferriss, Matt Mullenweg, Blake Mycoskie, Craig Nerenberg, and the Steven and Alexandra Cohen Foundation.

**Availability of data and materials:** Data will be made available on request.
